# Regulatory T cells in retroviral infections

**DOI:** 10.1371/journal.ppat.1006776

**Published:** 2018-02-15

**Authors:** Kim J. Hasenkrug, Claire A. Chougnet, Ulf Dittmer

**Affiliations:** 1 Rocky Mountain Laboratories, NIAID, NIH, Hamilton, Montana, United States of America; 2 Division of Immunobiology, Cincinnati Children's Hospital Medical Center, Cincinnati, Ohio, United States of America; 3 Institute for Virology, University Hospital Essen, University of Duisburg-Essen, Essen, Germany; University of Alberta, CANADA

## Abstract

Tight regulation of immune responses is not only critical for preventing autoimmune diseases but also for preventing immunopathological damage during infections in which overactive immune responses may be more harmful for the host than the pathogen itself. Regulatory T cells (Tregs) play a critical role in this regulation, which was discovered using the Friend retrovirus (FV) mouse model. Subsequent FV studies revealed basic biological information about Tregs, including their suppressive activity on effector cells as well as the molecular mechanisms of virus-induced Treg expansion. Treg suppression not only limits immunopathology but also prevents complete elimination of pathogens contributing to chronic infections. Therefore, Tregs play a complex role in the pathogenesis of persistent retroviral infections. New therapeutic concepts to reactivate effector T-cell responses in chronic viral infections by manipulating Tregs also came from work with the FV model. This knowledge initiated many studies to characterize the role of Tregs in HIV pathogenesis in humans, where a complex picture is emerging. On one hand, Tregs suppress HIV-specific effector T-cell responses and are themselves targets of infection, but on the other hand, Tregs suppress HIV-induced immune hyperactivation and thus slow the infection of conventional CD4^+^ T cells and limit immunopathology. In this review, the basic findings from the FV mouse model are put into perspective with clinical and basic research from HIV studies. In addition, the few Treg studies performed in the simian immunodeficiency virus (SIV) monkey model will also be discussed. The review provides a comprehensive picture of the diverse role of Tregs in different retroviral infections and possible therapeutic approaches to treat retroviral chronicity and pathogenesis by manipulating Treg responses.

## Author summary

Regulatory T cells (Tregs) play a very complex role in retroviral infections, and the balance of beneficial versus detrimental effects from Tregs can change between the acute and chronic phase of infection. Therefore, the development of therapeutics to treat chronic retroviral infections via modulation of Tregs requires detailed information regarding both the positive and negative contributions of Tregs in a particular phase of a specific infection. Here, we review the molecular mechanisms that initiate and control Treg responses in retroviral infections as well as the target cells that are functionally manipulated by Tregs. Basic findings from the Friend retrovirus mouse model that initiated this area of research are put into perspective with clinical and basic research from HIV studies. The targeted manipulation of Treg responses holds a bright future for enhancing immune responses to infections, vaccine responses, and for cure or functional cure of chronic retroviral infections.

## Regulatory T-cell responses in retroviral infections

Seminal experiments in 1995 proved the existence of a subset of T cells termed regulatory T cells (Tregs), with immunosuppressive properties critical for the control of autoimmune diseases [1]. Tregs have been demonstrated to suppress both the proliferation and function of effector T-cell subsets. They express the forkhead box protein 3 (Foxp3) transcriptional factor, which is the master regulator of the suppressive program (reviewed in [2]). In addition, Tregs generally express CD25, the high-affinity receptor for interleukin 2 (IL-2), which is essential for their development and maintenance [3–5]. Tregs have been subdivided into many subsets, but we will primarily discuss the two main subpopulations of Tregs, thymic Tregs (tTregs; previously called natural Tregs) [6], and peripherally derived Tregs (pTregs; previously called induced Tregs). tTregs arise as Foxp3^+^ Tregs directly from the thymus, are generally specific for self-antigens, require continuous antigenic stimulation for survival, and act to preserve self-tolerance [1, 7–9]. pTregs are converted to Foxp3-expressing Tregs from conventional CD4^+^ T cells in the periphery [10, 11] and thus are likely to be specific for a foreign antigen.

In addition to suppression of autoimmune reactivity, Tregs have also been shown to play an important role in immune evasion by cancer cells [12–14]. Therefore, the removal or blockage of Tregs is currently under investigation as a tumor therapy [14]. In 2001, experiments in mice infected with the mouse retrovirus Friend virus (FV) demonstrated for the first time that Tregs were also involved in infectious diseases [15], a finding that seemed paradoxical at the time. Subsequent studies demonstrated that Tregs were part of the normal immune response to pathogenic challenges with a number of various pathogens, including viruses, bacteria, and parasites (reviewed in [11, 16–18]). Such Treg responses are essential control mechanisms that appear to have evolved to prevent pathological damage from overly exuberant immune responses. The immunosuppressive activity of Tregs during infections both slows and dampens adaptive immune responses. For example, depletion of Tregs during acute FV infection doubles the number of virus-specific CD8^+^ T cells at the peak of infection and reduces viral loads by more than 10-fold [19]. Therefore, there is a trade-off between rapid and complete control of infection on one hand and minimizing inflammatory tissue damage on the other. An adverse consequence of Treg activity, especially suppression of the CD8^+^ T cell response, is the establishment and maintenance of chronic infection, as demonstrated in the FV model and suggested in HIV infection.

Kinetic studies in the FV model indicated that Tregs become activated and significantly expanded between one and two weeks post-infection (wpi) [20]. Interestingly, the expansion of CD4^*+*^ Tregs during FV infection is compartmentalized in tissues with high viral replication [21]. In those tissues (spleen, lymph nodes [LN], and blood), activated Tregs remain at high frequencies throughout the course of chronic FV infection, correlating with the presence of dysfunctional virus-specific CD8^+^ T cells [22]. In contrast, mouse livers contain relatively few Tregs with significantly greater proportions of functional CD8^+^ T cells and 10-fold less chronic infection [22].

The effect of HIV infection on the frequency of Tregs has been extensively studied. Human Tregs are usually defined as CD3^+^/CD4^+^/CD25^hi^/CD127^lo^/Foxp3^+^ T cells. In chronic, progressive HIV or simian immunodeficiency virus (SIV) infections, CD4^*+*^ Tregs are more frequent in the LN and gut-associated lymphoid tissue (GALT), where these viruses replicate most efficiently. Treg frequency correlates with viral loads and disease progression in HIV-infected individuals [23–26]. Treg increases occur early during the course of infection, as shown in the SIV monkey model [27]. Moreover, Tregs are activated during chronic HIV infection, with higher expression of molecules associated with activation, such as CD39 or cytotoxic T lymphocyte–associated protein 4 (CTLA-4) [23, 28, 29]. However, in contrast to FV infection, it should be noted that the overall number of Tregs decreases during chronic HIV infection, although Tregs remain selectively spared compared with other CD4 subsets [23, 29]. Highly active antiretroviral therapy (HAART) only partially abolishes the effect of HIV infection on abnormal Treg frequency and phenotypic characteristics [23, 29–31]. The effect of HIV infection on a subset of Tregs, the follicular regulatory T cells (Tfr), remains uncertain. These cells were first described in 2011 and control germinal center responses [32, 33]. Both human and murine Tfr display a unique transcriptional pattern overlapping that of both follicular T helper cells (Tfh) and Treg, notably coexpressing B cell lymphoma 6 protein (Bcl-6), Foxp3, and B lymphocyte–induced maturation protein 1 (Blimp-1). These C-X-C chemokine receptor 5 positive (CXCR5^+^) Tfr regulate the magnitude and character of the antibody (Ab) response by limiting the size of the Tfh compartment, inhibiting the selection of germinal center B cells, or both. Due to the high interest in the mechanisms regulating the development of broadly neutralizing Ab to HIV (reviewed in [34]), the function and homeostasis of these cells during HIV and/or SIV infection have been studied by several groups, but the data reported thus far are contradictory. Indeed, the frequency of LN or splenic Tfr was described in chronically SIV-infected rhesus macaques as decreased [35], unchanged [36], or increased [37] and as increased in HIV-infected individuals [38].

## Mechanisms of Treg expansion and/or accrual during retroviral infection

A critical issue in the study of Tregs is determining the mechanisms by which they become activated and expand during retroviral infections. Defining the molecular mechanisms of virus-induced Treg expansion might pave the way toward developing therapeutics to limit immunosuppressive Treg responses during infection. Such therapeutics might also be used to eliminate persistent infections. Conversely, the knowledge of how viruses induce Treg expansion could be used to therapeutically induce Tregs to treat autoimmune diseases or immunopathogenic responses.

### Virus-induced Treg expansion

Because Tregs express a normal T-cell receptor (TCR), it was originally thought that they might simply recognize and respond to viral antigens. That hypothesis, however, could not explain how or why the immune system would recognize one antigen as stimulatory and another as suppressive. In contrast with the theory of pathogen-specific recognition by Tregs, we were unable to demonstrate their presence in FV-infected mice despite many attempts using major histocompatibility complex (MHC) class II tetramers and TCR transgenic FV-specific CD4^+^ T cells [39, 40]. In fact, FV-specific TCRs are specifically excluded from the Treg repertoire in mice [39, 40]. Instead, FV-induced Tregs display a very broad distribution of TCR variable β (Vß) chain usage, suggesting that they recognize a wide variety of different self-antigens [39]. This is not too surprising because tTregs are generally specific for self-antigens, require continuous antigenic stimulation for survival, and act to preserve self-tolerance [1, 7–9]. Therefore, tTregs are quite different from conventional CD4^+^ T cells, which circulate and survive in a naïve state without TCR stimulation. tTregs are distinct from pTregs, which are converted to Foxp3-expressing Tregs from conventional CD4^+^ T cells in the periphery and may be virus specific [10, 11]. Virus-specific Tregs have been described in a few human infections, including HIV and hepatitis C virus (HCV), but they appear to be so infrequent in those infections that their biological relevance is questionable [41–44]. A side-by-side comparison of the TCR repertoires of Tregs versus conventional CD4^+^ T cells in HIV-infected individuals has not been done, although the analysis of purified Tregs from HIV-infected patients showed an overrepresentation of some Vα and Vβ families when compared to the Treg repertoire in uninfected individuals [45]. However, such overrepresentation does not appear to be Treg specific because it was also reported in unfractionated CD4^+^ T cells from HIV-infected individuals [46].

### Mechanisms of virus-induced Treg expansion

**Fig 1:**
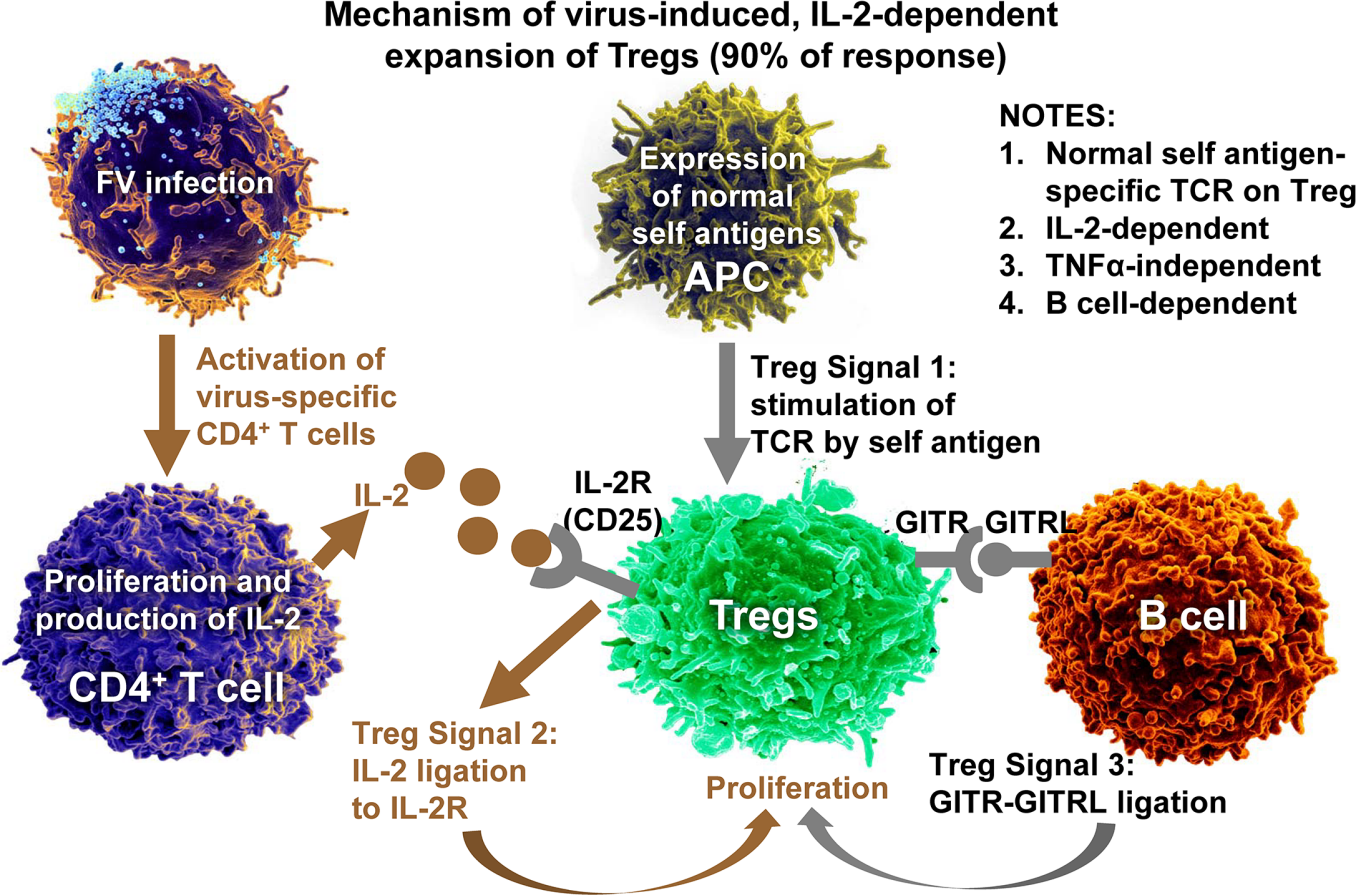
IL-2-dependent expansion of Tregs during FV infection. Depicted in brown are Treg activation events dependent on FV infection. No direct interactions between viral antigens and Tregs are required. Depicted in gray are required homeostatic signaling events for FV-induced Treg expansion. Homeostatic signaling levels are sufficient although FV infections may up-regulate expression of some membrane receptors. APC, antigen-presenting cell; FV, Friend virus; GITR, glucocorticoid-induced TNF receptor-related protein; GITRL, GITR ligand; IL-2, interleukin 2; TCR, T-cell receptor; TNFα, tumor necrosis factor α; Treg, regulatory T cell.

**Fig 2:**
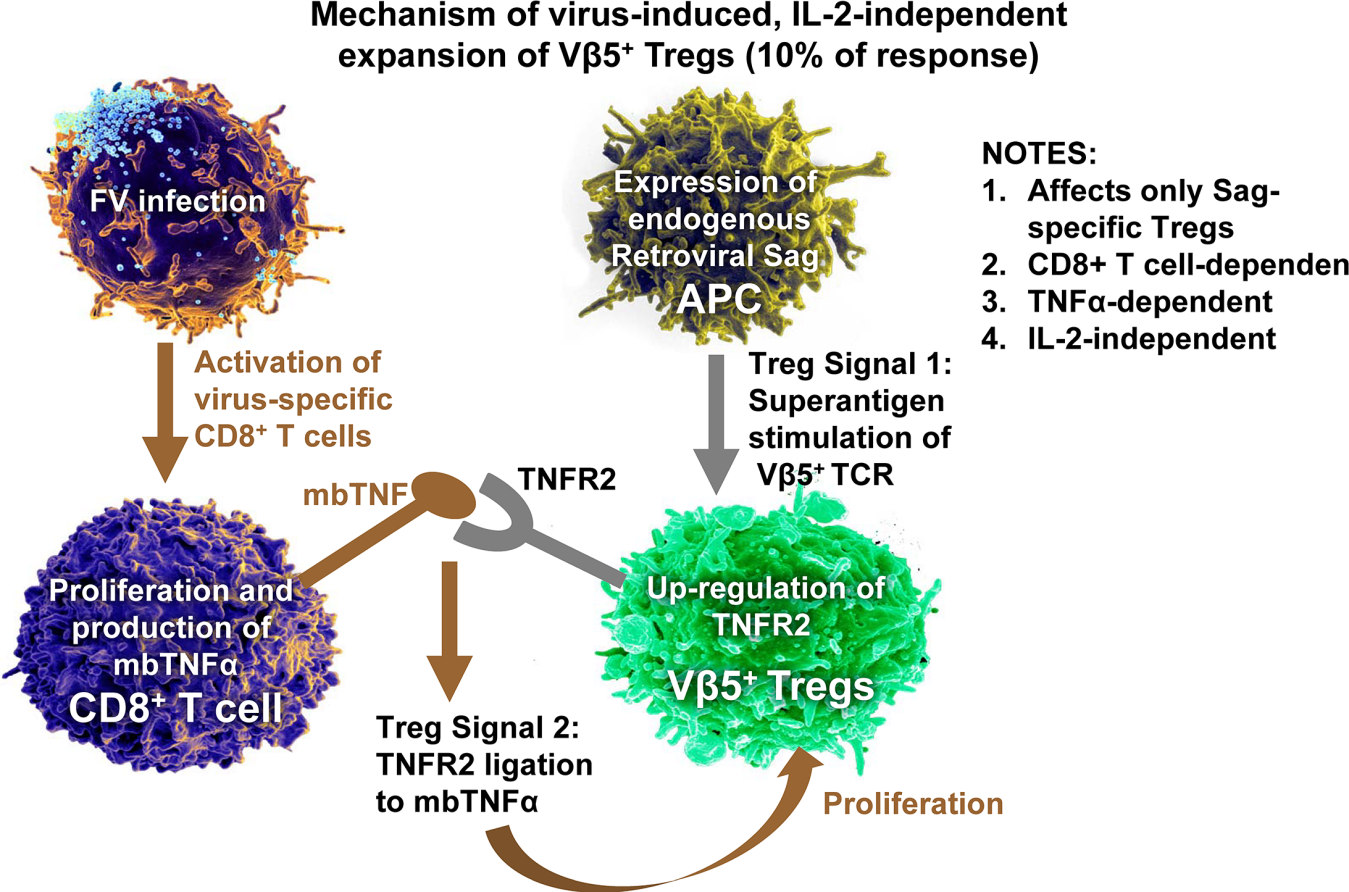
IL-2-independent expansion of Tregs during FV infection. Depicted in brown are Treg activation events dependent on FV infection. No direct interactions between exogenous viral antigens and Tregs are required. Depicted in gray are required homeostatic signaling events for FV-induced Treg expansion. FV infection indirectly up-regulates expression of TNFR2. FV, Friend virus; IL-2, interleukin 2; mb, membrane bound; Sag, superantigen; TNFR2, tumor necrosis factor receptor 2; Treg, regulatory T cell; Vβ5, T-cell receptor variable β chain 5.

Two distinct mechanisms of Treg expansion have been defined in the FV model, one IL-2-dependent (Fig 1) and the other IL-2-independent (Fig 2). Tregs express high levels of the IL-2 receptor (CD25), IL-2 is an essential differentiation factor for Tregs [47], and it is generally required for Treg function [3–5]. Therefore, it is not surprising that IL-2 is a required secondary signal involved in the expansion of most FV-induced Tregs [39]. In FV infections, IL-2 is predominantly produced by FV-specific effector CD4^+^ helper T cells responding to the infection [40]. It was recently shown that this IL-2-dependent Treg expansion is also dependent on interactions with B cells [48]. B cell–dependent Treg signaling occurrs via tumor necrosis factor (TNF) receptor superfamily member 18 (glucocorticoid-induced TNF receptor-related protein [GITR]) ligation with GITR ligand (GITRL) on B cells [48]. Of note, GITR–GITRL interactions are also required to control autoimmunity through regulation of Treg homeostasis [49]. The expanded Tregs in FV infection are tTregs as defined by their expression of the markers Foxp3, CD25, HELIOS, and Neuropilin 1 [39], as well as the fact that responding Tregs arise from preexisting tTreg populations and no conversion of conventional T cells into Tregs occurs [39].

A second mechanism of Treg induction, which is self-antigen specific but IL-2-independent, accounts for about 10% of the Treg expansion during FV infection (Fig 2) [39, 50]. Analyses of TCR Vß chain usage showed that a subpopulation of Tregs expressing the Vß5 chain of the TCR expanded disproportionately after FV infection [39]. This Treg subpopulation is specific for an endogenous retroviral superantigen (Sag) encoded by the mouse mammary tumor virus 9 (MMTV9). MMTV9 Sag binds to all CD4^+^ T cells expressing Vß5 chains and delivers a potent primary TCR signal that causes deletion of conventional CD4^+^ T cells during thymic selection in order to prevent autoreactivity [51, 52]. However, tolerogenic Foxp3^+^ Tregs are not deleted [53], and Sag stimulation results in the up-regulation of TNF receptor 2 (TNFR2) on the cell surface of Vß5^+^ Tregs in the periphery [50]. If TNFR2 on these Tregs binds the membrane-bound form of TNFα [50], it provides signal 2 for Treg activation. Interestingly, the membrane-bound form of TNFα is transiently up-regulated on recently activated effector CD8^+^ T cells, which in the case of FV infection are FV-specific CD8^+^ T cells [50]. Therefore, it is the effector CD8^+^ T cells, which eventually become the targets of Treg-mediated suppression, that provide the second signal for the activation and proliferation of Vß5^+^ Tregs. Apparently, the combination of a potent Sag signal combined with TNFR2 signaling is strong enough to negate the normal requirement for IL-2. This IL-2-independent mechanism does not appear restricted to FV infection because the Vß5 subset of Tregs also disproportionately expands in mice persistently infected with lymphocytic choriomeningitis virus (LCMV) [54]. Treg subpopulations in humans also express TNFR2 [55–57], but it remains to be determined whether Tregs that are specific for endogenous retroviral antigens also exist in humans. Along this line, it is interesting that human endogenous retroviral (HERV)-specific conventional T cells expand in HIV-infected individuals [58].

Mechanisms promoting the expansion of Tregs during HIV infection are not clearly understood, but it appears to be more a relative sparing than a real expansion, i.e., fewer CD4^+^ Tregs are killed by HIV than conventional CD4^+^ T cells. The field has not yet come up with a satisfactory explanation of why absolute numbers of Tregs decrease even though, based on all the mechanisms studied so far, an expansion would be expected. First, ex vivo and in vitro studies suggest that the proportion of pTregs may increase during HIV infection. One potential mechanism for this enhanced conversion is that HIV or SIV infection induces semimature dendritic cells (both myeloid and plasmacytoid DCs) that have been shown to enhance Treg differentiation from conventional CD4^+^ T cells [59, 60]. In vitro culture with myeloid DCs from HIV-infected individuals also promotes Treg expansion [61]. Virus-infected DCs are also involved in Treg expansion in FV infection, although there is no conversion of conventional T cells into pTregs [62]. Second, Treg proliferation appears augmented during chronic HIV infection, as Tregs from chronically infected patients express higher levels of Ki-67 than those from uninfected individuals [29, 63]. Finally, Tregs also seem less prone to HIV-induced apoptosis [26, 64, 65], which is consistent with the fact that Tregs express lower levels of proapoptotic molecules than their conventional CD4^+^ T-cell counterparts in the gut of SIV-infected rhesus macaques [66]. However, all these ideas are based on ex vivo or in vitro studies because the tools to ascertain whether these pathways are operational in vivo are lacking in humans and nonhuman primates.

## Targets of Treg suppression during retrovirus infection

### CD8^+^ T-cell targets

CD8^+^ T cells are extremely potent effector cells that not only secrete potent inflammatory cytokines but also kill infected cells through the release of cytotoxic granules, including perforin and granzymes. Therefore, they have the potential to cause significant collateral damage during a host immune response and were the first antiviral cells recognized as targets for Treg-mediated suppression [15, 67]. Studies using adoptively transferred TCR-transgenic, FV-specific CD8^*+*^ T cells [67], as well as studies in which mice could be selectively depleted of Tregs [19, 21], indicated that Tregs begin to suppress CD8^+^ T-cell proliferation and effector functions during the late phase of acute FV infection [21]. Tregs mainly affect the exocytosis pathway of CD8^+^ T-cell killing rather than the first apoptosis signal receptor (Fas; or CD95)/Fas ligand (FasL) pathway [68]. Treg-mediated suppression is maintained during chronic FV infection and contributes to the exhausted phenotype of CD8^+^ T cells [69]. Therefore, selective depletion of Tregs during chronic FV infection reactivates residual FV-specific CD8^+^ T cells to secrete multiple cytokines, produce cytotoxic granules, and develop in vivo cytotoxicity resulting in significantly reduced chronic viral set points [69]. Interestingly, the chronic exhaustion of FV-specific CD8^+^ T cells is also influenced by the expression of inhibitory receptors [70, 71], a separate immune checkpoint mechanism that can act independently of Treg responses [70, 71]. Importantly, the suppressive activity of FV-induced Tregs on CD8^+^ T cells is not antigen specific. After becoming activated and expanded during FV infection, Tregs can suppress ovalbumin-specific CD8^+^ T cells or mixed lymphocyte reactions in vitro [15, 72], and they also impair mouse CMV-specific T-cell responses in vivo [73].

**Fig 3:**
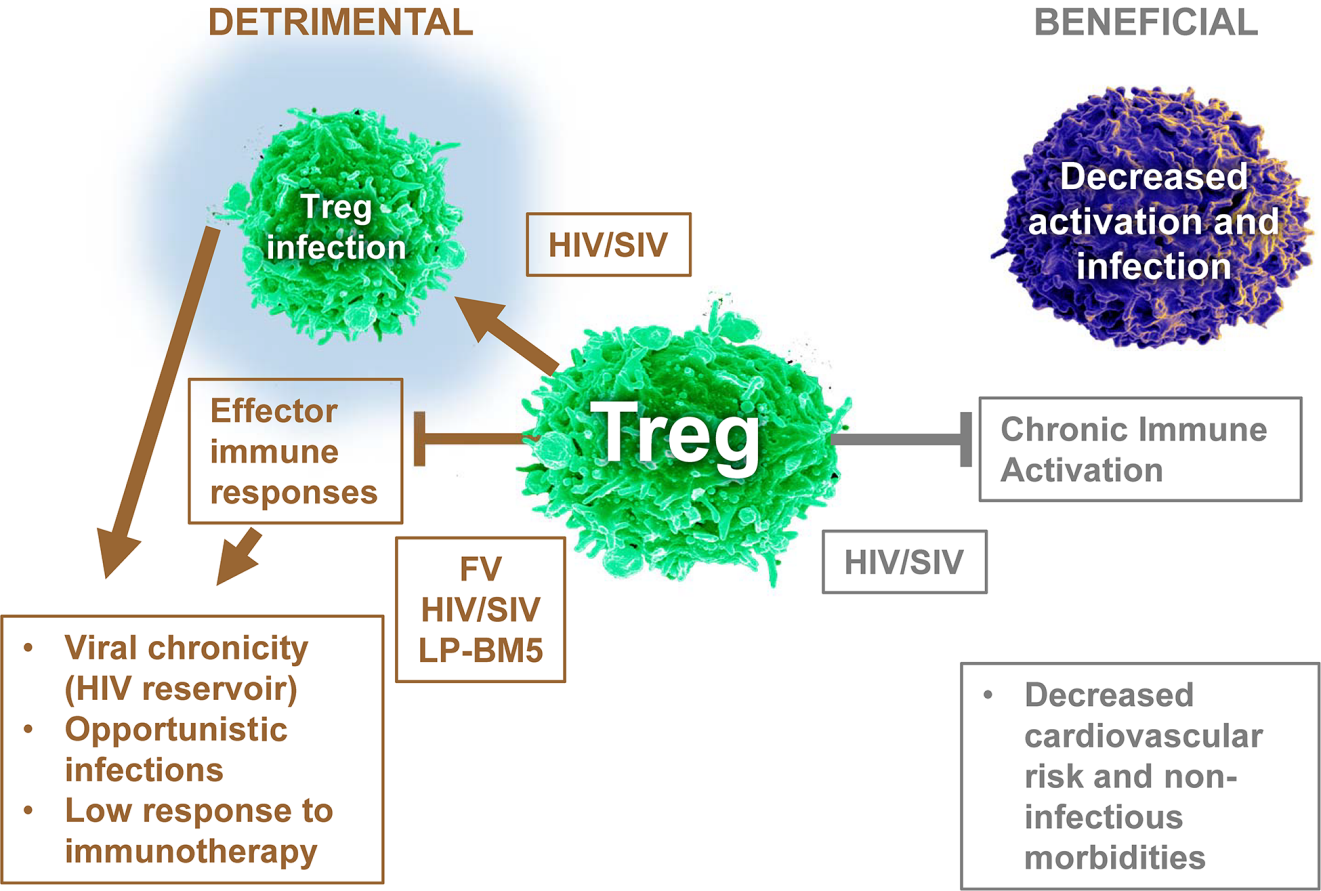
Duality of Treg effects in retroviral infections. Deterimental effects are depicted in brown, and beneficial effects are depicted in gray. Arrows indicate effects, while blocked lines indicate blocked activities. FV, Friend virus; LP-BM5, murine retrovirus LP-BM5; SIV, simian immunodeficiency virus; Treg, regulatory T cell.

There is substantial experimental evidence that both Tregs and inhibitory receptor expression play key roles in T-cell exhaustion and immune dysfunction during chronic HIV and SIV infections [70, 74–76]. Early in vitro studies using Tregs from HIV-1^+^ patient samples showed that both HIV- and CMV-specific CD8^+^ T-cell responses were suppressed [77–79]. Importantly, in vivo studies have also illustrated Treg effects on CD8^+^ T cells. For example, studies done by the Apetrei-Pandrea group used the human IL-2/diphtheria toxin fusion protein (Ontak) to deplete Tregs in SIV-infected controller macaques [80]. Following this treatment (leading to a >75% loss in Treg proportion), major CD4^+^ T-cell activation occurred, leading to the reactivation of latent SIV. However, Treg depletion also significantly boosted SIV-specific CD8^+^ T-cell frequencies, resulting in the rapid clearance of reactivated virus. These data demonstrate the complex in vivo role of Tregs in controlling SIV-specific immune responses. These results also support the concept that the early emergence and persistent accrual of Tregs in LN during pathogenic SIV and/or HIV infection likely impairs the protective antiviral CD8^+^ T-cell response, as suggested by previous associative studies (Fig 3) [26, 27, 81]. Also of interest in this context is the fact that CD8^+^ T cells restricted by the human leukocyte antigen (HLA) allele groups associated with delayed HIV disease progression (notably HLA-B*27 and HLA-B*57) were not suppressed ex vivo by Tregs [82]. This resistance to suppression implies that Treg accrual plays a role in HIV-1 disease progression by hampering CD8^+^ T-cell responses. As described in FV infection, Treg functions are not virus specific in HIV infection because they also control CD8^+^ T-cell responses against other viruses, such as CMV [31, 83]. Therefore, their increased frequency likely contributes to the development of AIDS-associated infections in untreated patients. Accordingly, a higher percentage of circulating CD4^+^/FOXP3^+^ Tregs was shown to be predictive of CMV end-organ disease [84].

### CD4^+^ T-cell targets

Effector CD4^+^ T cells, responsible for providing help for effector B and T-cell responses and the development of immunological memory, are also targeted by Tregs in the FV model. Tregs can suppress the proliferation and cytokine production by type I helper T cells during FV infection [85]. In addition, they also control the cytotoxicity of virus-specific CD4^+^ T cells during acute FV infection but only in circumstances in which CD8^+^ T cells are absent. If cytotoxic CD8^+^ T cells are present, Treg depletion does not result in a substantial induction of CD4^+^ T-cell cytotoxicity [86]. However, in acutely FV-infected mice lacking both CD8^+^ T cells and Tregs, a massive expansion of cytotoxic CD4^+^ T cells occurs. These CD4^+^ T cells can kill FV antigen-labeled targets in an MHC class II–dependent exocytosis process. Such CD4^+^ T cell–mediated cytotoxicity appears to be a mechanism to compensate for the lack of CD8 functionality in chronically FV-infected mice [87]. In chronic FV infections in which CD8^+^ T cells are dysfunctional, cytotoxic CD4^+^ T cells take over and kill virus-infected targets in a Fas/FasL-mediated pathway [88].

During HIV/SIV infection, Tregs decrease CD4 functionality, inhibiting both HIV-specific and polyclonal responses (reviewed in [89–91]). However, the role of Tregs is complex (Fig 3) because low Treg frequency during HIV infection is associated with increased immune activation [92–94], and such activation facilitates HIV infection of target cells. Therefore, Tregs also limit virus spread. This has been shown by in vivo Treg depletion of SIV-infected controller macaques as mentioned above [80] and also in the nonpathogenic model of SIV infection in African Green Monkeys [95]. It has also been shown in vitro that Tregs limit the HIV infection of activated conventional CD4^+^ T cells [96], macrophages [97], and DCs [98].

Tfr, which suppress Tfh necessary for B cell help in germinal centers [99] and subsequent Ab responses [100, 101], have been the subject of several recent studies [34, 102, 103]. Tfr normally differentiate from tTregs rather than conventional antigen-specific T cells [104], although exceptions have been observed [105]. The frequency of Tfr in LN is inversely correlated with the frequency of Tfh and germinal center B cells in LN [35, 36], and also with the avidity of plasma Abs recognizing SIV envelope proteins [35]. Ex vivo HIV infection of human Tfr increased their expression of molecules associated with regulatory activity, namely CTLA-4, lymphocycte activation gene 3 (LAG-3), GITR, and galectin-3, and also enhanced production of IL-10 and transforming gowth factor β (TGF-β). Consequently, these infected Tfr are very efficient at inhibiting Tfh proliferation and Tfh production of cytokines for B cell help [37]. Similarly, transcriptional analysis of LN Tfr after SIV infection revealed a profile of increased activation [36]. In the same line, HIV-1-infected individuals with broadly neutralizing Abs had a lower frequency of Tregs and a higher frequency of circulating memory Tfh compared with those who did not develop such Abs [106]. This increased response was not specific for HIV antigens because the individuals with broadly neutralizing Abs also had a higher frequency of auto-Abs [106].

### Natural killer cell targets

Natural killer (NK) cells are important innate lymphoctyes that produce proinflammatory cytokines and can kill tumor cells and infected cells following their activation. It has been shown that Tregs indirectly regulate NK cell maturation by restraining IL-2 availability [107]. Therefore, Tregs can regulate the ability of NK cells to react to missing self-antigens on target cells, essentially acting as an IL-2 sink [108]. In a related manner, Tregs regulate NK responses during FV infection. Normally, NK cell responses to acute FV infection are rather weak, and NK cells contribute only marginally to virus control [109]. However, a large part of this impotence is because FV-induced Tregs suppress NK cell proliferation, maturation, and effector cell differentiation during the acute phase of FV infection [110]. Because Tregs express high levels of the high-affinity IL-2 receptor CD25 while NK cells only express the low-affinity IL-2 receptor CD122, Tregs can outcompete NK cells for IL-2 consumption. This lack of IL-2 availability reduces the activation and differentiation of NK cells in FV-infected mice. When Tregs are depleted or IL-2 is experimentally directed to the CD122 receptor of NK cells, full activation of NK cells and significant anti-FV activity are observed. These results indicate that targeted immunotherapy can abrogate the suppression of NK cells by Tregs and enhance virus control. To date, nothing has been published about the influence of Tregs on NK cell responses in HIV-infected humans.

### Antigen-presenting cell targets

Tregs also mediate their suppressive action by acting directly on antigen-presenting cells, such as DCs, decreasing DC maturation and subsequently T-cell activation [111]. The formation of Treg–DC conjugates in vivo and in vitro also suggests that DCs may be primary targets of Treg suppression [111–115]. Because DCs facilitate HIV dissemination to the lymphoid organs by enabling HIV infection of CD4^+^ T cells [116, 117], we analyzed how Tregs affect viral transmission from DC to effector T cells. In an in vitro model, Tregs significantly decreased HIV transmission from DC to T cells, notably impairing actin polymerization and the trafficking of HIV viral particles to the immunological synapse [98].

### Myeloid-derived suppressor cells

Using mice infected with the immunodeficiency-inducing retroviral complex known as LP-BM5, the Green lab demonstrated an increased proportion of both IL-10-producing Tregs and immunosuppressive myeloid-derived suppressor cells (MDSCs) [118]. In adoptive transfer experiments, it was further shown that these two different immunosuppressive cell subsets reciprocally modulated each other’s function. MDSCs modulated IL-10 production by Tregs, and in the absence of Tregs, MDSC suppression of T cells was increased.

## Effect of HIV infection on Treg functionality

Because Tregs are themselves susceptible to HIV infection (Fig 3) [119], one question that has been addressed by several groups is whether their functionality, on a per cell basis, was altered by HIV infection. Of note, bulk Tregs isolated from chronic HIV-1 progressors are as functional as those from uninfected individuals or HIV controllers [94, 120, 121]. However, binding of inactivated HIV or HIV glycoprotein 120 (gp120) to CD4 on Tregs enhanced not only their survival but also their suppressor activities [26, 64, 65]. In contrast, one study reported that productively infected Tregs were less functional on a per cell basis than uninfected, but HIV-exposed, Tregs [122]. Therefore, there may be a different effect from productive infection compared to exposure to defective HIV particles circulating during HIV infection.

## Mechanisms of suppression

Knowledge about the molecular mechanisms underlying Treg-mediated suppression of retrovirus-specific T-cell responses is limited. In FV infection, suppression occurs in a direct cell-to-cell contact–dependent manner independently of the presence of antigen-presenting cells [72]. Immunosuppressive cytokines such as IL-10 and TGF-β secreted by CD4^*+*^ Tregs did not contribute to Treg-mediated immunosuppression in either in vitro or in vivo experiments [67, 72]. Furthermore, Tregs that respond to FV infection do not secrete granzymes, excluding granzyme-mediated killing of effector T cells [123]. This is in line with findings that Tregs inhibit effector T-cell proliferation and function [21] but do not induce apoptosis in T cells during FV infection. The exact mechanism of suppression by Tregs in FV-infected mice is still under investigation. Tregs have been reported to control T-cell activation and proliferation via a contact-dependent mechanism involving cyclic andenosine monophosphate (cAMP) [124], a mechanism that might also be operative during FV infection.

During HIV infection, CTLA-4 (CD152), CD39, and cAMP have been intensively studied as mechanisms of suppression. CTLA-4 coinhibitory molecules are expressed by Tregs from HIV-infected individuals at higher levels than in uninfected individuals [23]. CTLA-4 blockade early during SIV infection of rhesus macaques led to an increase in T-cell activation and viral replication [125]. However, interpretation of these data must be cautious because the Treg role was not specifically addressed in this study. Moreover, Kaufmann et al. showed that Tregs did not play a major part in the in vitro CTLA-4-mediated inhibition of HIV-specific responses because CTLA-4 blockade was still operative in the absence of Tregs and most HIV-specific CTLA-4^+^/CD4^+^ T cells were not Tregs [126].

The proportion of CD39^+^ Tregs also increases during chronic HIV infection [23, 28, 29], and in vitro blockade of CD39 reverses Treg suppression of HIV-specific CD8^+^ T cells [28]. This suppressive mechanism may be particularly important in vivo because both CD4^+^ and CD8^+^ T cells from chronically HIV-infected individuals express high levels of the adenosine A2A receptor CD39 [28, 127]. Importantly, a *CD39* gene polymorphism leading to low CD39 expression is associated with a slower progression to AIDS [28]. However, this association cannot be solely ascribed to CD39^+^ Tregs because type 1 regulatory T cells (Tr1) or CD8^+^ regulatory cells also express CD39, and both cell types are frequent in HIV-infected individuals [128, 129].

As mentioned above, cAMP also participates in Treg suppression. Upon stimulation with HIV gp120, human Tregs were shown to accumulate cAMP in their cytosol. Furthermore, the tolerizing effect of HIV gp120 in a xenogeneic graft-versus-host disease model was strictly dependent on the induction of cAMP in human Tregs [64]. In agreement with this hypothesis, Tregs from HIV-1-infected individuals express high levels of intracellular cAMP [127]. We also showed that Treg cAMP was important to control HIV replication in activated effector T cells because suppression was abolished by chemically decreased cAMP levels in Tregs. A similar effect was found in DCs [98]. Blocking gap junction formation between Tregs and effector T cells and inhibiting protein kinase A in effector T cells also abolished Treg suppression, indicating a requirement for cell-to-cell contact [96].

## Treg immunotherapy in lentiviral infections: The pros and cons

Because Tregs can blunt effector immune responses during retroviral infections, it is important to determine whether Treg responses can be manipulated in vivo to overcome suppression and induce immunity. Several approaches have been studied in the FV model. As mentioned earlier, Tregs express GITR, and treatment of mice during acute FV infection using blocking anti-GITR Ab significantly increased virus-specific CD4^+^ and CD8^+^ T-cell numbers and function, reduced pathology, and produced long-term increases in CD8^+^ T-cell functionality [130]. A caveat to this study is that all of the effects could not be attributed to Tregs but might also have been direct activation of the CD8^+^ T cells. In another study, it was shown that CD8^+^ T cells could be rendered resistant to Treg-mediated immunosuppression by stimulating them with an agonistic Ab specific for the CD137 (4-1BB) costimulatory molecule [131]. Interestingly, this CD137 agonistic Ab could also be used to reprogram Tregs to become cytotoxic CD4^+^ T cells with antitumor activity [132]. In those studies, the reprogrammed cells expressed the T-box transcriptional factor Eomesodermin and granzyme B without loss of Foxp3 expression.

Treg responses have also been successfully manipulated to enhance the efficacy of therapeutic vaccines during chronic FV infection. Therapeutic vaccination of chronically FV-infected mice with functionalized calcium phosphate (CaP) nanoparticles temporarily reactivated cytotoxic CD8^+^ T cells and significantly reduced viral loads [133]. Transient ablation of Tregs during this nanoparticle-based vaccination strongly enhanced antiviral immunity and further decreased chronic viral set points [134]. In the context of HIV infection, Tregs also seem detrimental to the efficacy of therapeutic vaccines [135, 136], suggesting that adding a Treg blocker along with the vaccine might lead to higher clinical benefit although, to our knowledge, this strategy has not yet been tested.

During HIV infection, another exciting prospect is that Treg manipulation could be used to purge the HIV reservoir. In the last 15 years, it has become evident that even the most highly efficient antiretroviral therapy will not cure HIV, due to the persistence of integrated, replication-competent proviruses within host cellular DNA (rev. in [137]). Therefore, developing “shock and kill” strategies has emerged as a priority in the field of HIV research. The underlying concept of these strategies is that if it were possible to induce viral expression from the latent reservoir, then it might be feasible to trigger immune-mediated clearance of the infected cells through CTLs, NK cells, or immunotoxins. However, a critical limitation of these strategies is that effective and safe latency reversing agents have not yet been identified, and exhaustion of the HIV- and/or SIV-specific CTLs hampers their capacity to properly eliminate reactivated virus (rev. in [137]). In this context, manipulation of Treg numbers and/or functions could be advantageous, based on the results of Treg depletion in simian models. Indeed, as mentioned above, transiently decreasing Treg numbers by Ontak treatment led to both reactivation of latent HIV and boosted SIV-specific CD8^+^ T-cell frequencies in virally suppressed rhesus macaques. The result was rapid clearance of the reactivated virus [80], thus achieving both goals of the “shock and kill” approach at the same time. This treatment was safe, without signs of untoward autoimmune disease ([80] and 2017 discussion between Drs Apetrei, Pandrea, and Chougnet). Transient Treg ablation might be more efficacious in this context than the manipulation of Treg function because Tregs themselves have been suggested to be a reservoir for latent HIV [138, 139], although that has not been confirmed by all studies [140]. Of note, in an HIV-infected patient being treated for melanoma with Ipilimumab, an anti-CTLA-4 Ab known to effect Tregs, transient decreases in HIV-1 RNA were detected following infusions of the Ab [141].

Besides HIV, Treg-mediated suppression of antiviral effector cells is a matter of concern in other chronic virus infections such as HCV, hepatitis B virus (HBV), human papillomavirus (HPV), and Epstein-Barr virus (EBV) [74] and limits responses to therapeutic vaccines in patients infected by these chronic viruses. Notably, patients with large lesions due to HPV-induced vulvar intraepithelial neoplasia also had high frequencies of HPV-specific CD4^+^/CD25^+^/Foxp3^+^ T cells and displayed a lower HPV-specific interferon γ (IFNγ)/IL-10 ratio after therapeutic vaccination [142]. Therefore, therapeutic manipulation of Tregs to reactivate or enhance virus-specific immunity and subsequently reduce chronic infection levels could have widespread clinical applications.

In considering therapeutic manipulation of Tregs, the Janus nature of Tregs during HIV infection should not be forgotten. Tregs limit generalized immune activation during HIV infection [92–94], and in the era of suppressive HAART, persistence of immune activation is highly associated with the noninfectious causes of HIV-driven increased mortality (rev. in [143] and [144]). Relevant to this concept, low Treg frequency in HIV elite controllers is strongly associated not only with immune activation but also with accelerated atherosclerosis and other morbidities linked to inflammation [145, 146]. Therefore, depletion or ablation of Treg function to boost immune responses or purge the reservoir may end up producing deleterious consequences. In this context, increasing Treg frequency could be beneficial to HIV patients because Tregs limit atherosclerosis (rev. in [147, 148]). The ongoing statin clinical trials in HAART-treated patients will be informative in this context. Statins are given to HAART-treated patients, including normolipidemic patients (REPRIEVE Phase IV clinical trial), with the goal of decreasing HIV-associated cardiovascular risk. Importantly, we and others have shown that statin treatment increases Treg frequency [149, 150], which likely contributes to their pleiotropic anti-inflammatory properties. Analysis of Treg dynamics in statin-treated HIV-infected patients, in relation to immune activation and clinical outcome, may provide important information about the exact role of Tregs in HIV infection.

## Conclusions

Given the complex roles that Tregs play in retroviral and other infectious diseases, the successful application of therapeutics to treat infectious diseases via modulation of Tregs will obviously require extremely detailed information regarding both the positive and negative contributions of Tregs in a particular infection. We now know that the balance of beneficial versus detrimental effects from Tregs can change during the course of a retroviral infection, especially between the acute and chronic phase of infection. Therefore, it is important to recognize not only which infectious agent induced the Treg response but also the phase of the infection. The discovery of molecular mechanisms that initiate and control Treg responses in infectious diseases is key to the understanding and manipulation of these complex processes. For example, the presence of endogenous retroviruses that express specific antigens may strongly affect individual Treg responses. We also know that virus-induced Tregs alter not only the response against the virus that initiated the response but also subsequent responses to other infections. Therefore, the patient’s history of infections can affect the homeostatic level of Treg-mediated suppression. Numerous other factors that need further study may also play important roles in Treg responses, including sex, age, underlying medical conditions, drug use, stress, etc. It is extremely important that further investigations continue to delineate the important factors influencing the impact of Tregs on both pathology and antiviral immunity in retroviral infections so that personalized medicine can be developed. Discovery of the specific mechanisms that Tregs use for immunosuppression may even allow differential blockade of detrimental functions while maintaining beneficial functions. The targeted manipulation of Treg responses holds a bright future for treating not only autoimmune diseases but also in enhancing vaccine responses, immune responses to infections, and in eradication of chronic infections.
